# Assessment of TiO_2_ Blocking Layers for Cu^II/I^-Electrolyte Dye-Sensitized Solar Cells by Electrochemical Impedance Spectroscopy

**DOI:** 10.1021/acsaem.1c03433

**Published:** 2022-02-11

**Authors:** Hannes Michaels, Marina Freitag

**Affiliations:** † Department of Chemistry - Ångström Laboratory, Uppsala University, Box 523, Uppsala 75120, Sweden; ‡ School of Natural and Environmental Science, Newcastle University, Newcastle upon Tyne NE1 7RU, United Kingdom

**Keywords:** dye-sensitized solar cells, impedance spectroscopy, blocking layer, solar cells, spray pyrolysis, electrochemical double layer, electrode capacitance, recombination

## Abstract

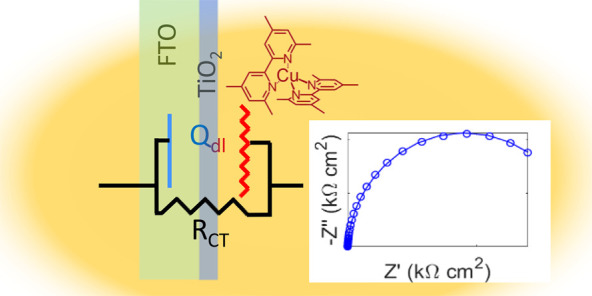

The TiO_2_ blocking layer in dye-sensitized solar cells is the most difficult component to evaluate at thicknesses below 50 nm, but it is crucial for the power conversion efficiency. Here, the electrode capacitance of TiO_2_ blocking layers is tested in aqueous [Fe(CN)_6_]^3–/4–^ and correlated to the performance of photoanodes in devices based on a [Cu(tmby)_2_]^2+/+^ electrolyte. The effects of the blocking layer on electronic recombination in the devices are illustrated with transient photovoltage methods and electrochemical impedance analysis. We have thus demonstrated a feasible and facile method to assess TiO_2_ blocking layers for the fabrication of dye-sensitized solar cells.

## Introduction

**Figure 1:**
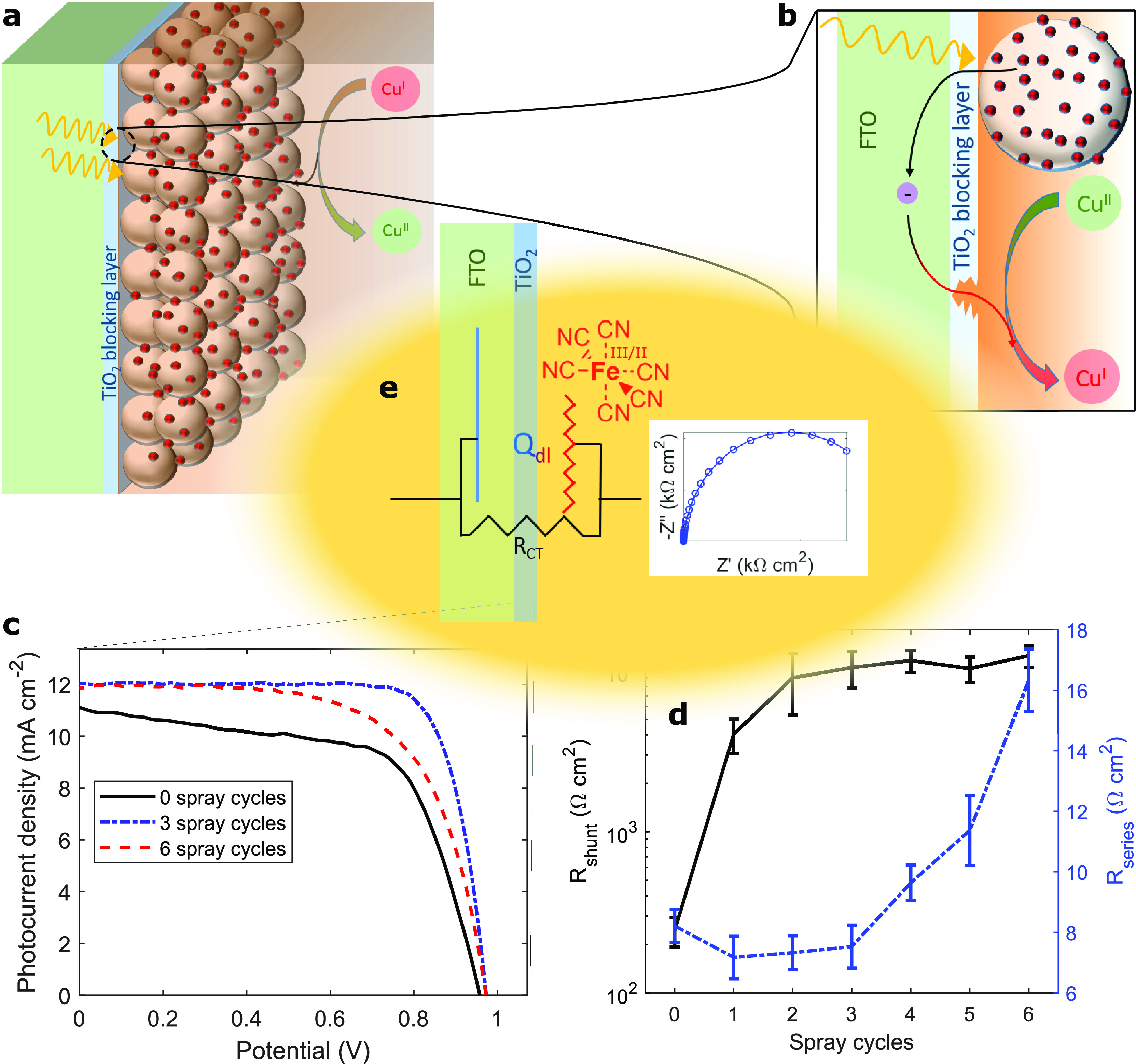
Importance of the TiO_2_ blocking layer in dye-sensitized solar cells. (a) Scheme of a DSC photoanode. (b) Deficiencies in the blocking layer cause electronic recombination with the electrolyte. (c) Too thin or too thick blocking layers cause transport or recombination losses in DSC performance. One spray iteration in our experiment corresponded to the deposition of 0.21 mL of titanium bis(isopropoxide) bis(acetylacetonate) precursor per 100 cm^2^ substrate area. (d) Shunt and series resistance of DSCs by blocking layer thickness. (e) Proposed quantification of the blocking layer via its Helmholtz capacitance.

Dye-sensitized solar cells (DSCs) present a low-cost, alternative photovoltaic technology, appealing in their modularity, colorfulness, transparency, and, recently, photochromaticity.^1−3^ Their first description by Gerischer^4^ and realization by O’Regan and Grätzel^5^ in 1991 sparked massive research efforts across all disciplines of materials science.^6^ In DSCs, molecular dyes are anchored on semiconductor nanoparticles (Figure 1a). Upon absorption of photons, charge carriers are injected into the semiconductor. The dye molecules are regenerated by a redox electrolyte or solid-state hole transport material.^7,8^ Given their high performance in low and indoor lighting conditions, DSCs have recently shown promise to make smart and autonomous sensors for the Internet of Things independent from other external power sources.^9−11^

Especially in environments of low illuminance, however, excited and into the photoanode semiconductor injected electrons must be carefully protected from recombination with the oxidized redox species in the electrolyte or hole transport material. One common procedure in this endeavor is the deposition of a TiO_2_ dense layer, or *blocking* layer, onto the anode substrate. The blocking layer inhibits electronic recombination at the electrode by keeping the redox mediator at a distance from the FTO electrode (Figure 1b).^12−15^ If applied too thick, incomplete charge collection occurs, and fill factor losses (Figure 1c) are the consequence of increased series resistance arising in the device (Figure 1d).

At thicknesses below 50 nm, the TiO_2_ blocking layer is the component in DSC devices that is most difficult to evaluate but at the same time critical for their power conversion efficiency.^16−18^ Despite having been introduced over two decades ago,^19^ reproducible deposition, between batches as well as between laboratory members, is oftentimes challenging. The deposition of blocking layers is the first step in the process of manufacturing DSC devices, and it is followed by several sintering and fabrication steps. Often does one loop of feedback on the blocking layer deposition take multiple days to a week, making measures of quality control on the TiO_2_ blocking layers extremely tedious and time-consuming.

The electrochemical properties of TiO_2_ blocking layers in the frequency domain have been the subject in a small number of previous studies. Cameron et al. as well as Góes et al. probed the blocking properties of TiO_2_ dense layers in conjunction with the I^–^/I_3_^–^ electrolyte.^20,21^ Grätzel and co-workers highlighted how the choice of the deposition method between spray pyrolysis, electrochemical bath deposition, ALD, or thermal oxidation of Ti causes different types of defects in the blocking layers, as such with altered sensitivity to different-sized redox species.^22^ Nevertheless, direct advice on how to quantify the blocking properties of TiO_2_ dense layers to be used in conjunction with the comparably recently introduced, and unfortunately recombination-prone, copper electrolytes is still lacking.

Here, we demonstrate a systematic methodology for directly assessing and controlling the electronic properties of TiO_2_ blocking layers. The electronic capacitance of TiO_2_ blocking layers in aqueous [Fe(CN)_6_]^3–/4–^ (Figure 1e) directly correlates to the blocking layers’ ability to inhibit recombination in a DSC device. Electronic recombination processes at different locations within the DSC devices, depending on blocking layer thickness, are then highlighted by transient photovoltage and electrochemical impedance analysis.

Our here-introduced methodology will serve a wide range of electrochemists eyeing to directly evaluate the electronic properties of carrier-selective dense layers, from DSC blocking layers to their analogues in perovskite solar cells and other thin-film technologies, as well as potentially their hole-selective p-type semiconductor counterparts.

## Experimental Section

### Materials

Unless otherwise stated, all materials were purchased from commercial suppliers and used without further purification; potassium ferrocyanide (K_4_Fe(CN)_6_), potassium ferricyanide (K_3_Fe(CN)_6_), lithium iodide, iodine, lithium bis(trifluoromethanesulfonyl)imide, tetrabutylammonium hexafluorophosphate, and dry acetonitrile from Sigma-Aldrich; conductive glass substrates (TEC 15) from Pilkington; 1,2-dimethyl-3-propylimidazolium iodide and *N*-methyl benzimidazole from TCI; Cu(tmby)_2_TFSI_1;2_ and Co(bpy)_3_(PF_6_)_2;3_ complexes as well as the Y123 dye from Dyenamo (Stockholm).

### TiO_2_ Blocking Layer Preparation

The TiO_2_ blocking layers were deposited onto cleaned (RBS solution, water, ethanol, UV-ozone) Nippon sheet glass (Pilkington), with 10 Ω sheet resistance. The dense layers of TiO_2_ deposited via spray pyrolysis were heated to 450 °C on a hot plate; then, a 0.2 M titanium bis(isopropoxide) bis(acetylacetonate) solution in isopropanol was sprayed onto the glass substrates by manual repetition of spray passes over the substrates, between which the substrates were let rest for 1 min. One spray iteration in our experiment corresponded to the deposition of 0.21 mL of precursor per 100 cm^2^ substrate area. Alternatively, the blocking layers were prepared from chemical bath deposition from a 40 mM aqueous solution of titanium tetrachloride at 70 °C and subsequent annealing at 450 °C.

### Solar Cell Preparation

Onto the prepared TiO_2_ blocking layers, 0.384 cm^2^ (7 mm diameter circles) TiO_2_ photoanodes were screen-printed (Seritec Services SA, Corseaux, Switzerland) from DSL 30 NRD-T (Dyesol/GreatCellSolar, Queanbeyan, Australia) colloidal (30 nm) TiO_2_ paste (4 μm). After brief drying at 120 °C, a scattering layer (Dyesol/GreatCellSolar WER2-0, 400 nm) was screen-printed onto of the mesoporous film (4 μm), followed by gradual heating toward a 30 min sintering step at 500 °C. The substrates were post-treated with a 13 mM aqueous TiCl_4_ solution for 30 min at 70 °C and then sintered again at 450 °C for 30 min. After cooling, the titania films were immersed into the sensitizer solution of 0.1 mM Y123, 0.2 mM chenodeoxycholic acid in a volumetric acetonitrile:*tert*-butanol mixture for 16 h. PEDOT counter electrodes were manufactured via electropolymerization of 3,4-ethylenedioxythiophene from a 0.01 mM aqueous solution with 0.1 M sodium dodecyl sulfate.^23^ The redox electrolyte solution was prepared with 0.2 M Cu(tmby)_2_TFSI and 0.04 M Cu(tmby)_2_TFSI_2_, 0.1 M lithium bis(trifluoromethanesulfonyl)imide, and 0.6 M *N*-methyl benzimidazole in acetonitrile. Cells were assembled by using ThreeBond (Dusseldorf, Germany) 3035B UV glue and cured with a CS2010 UV source (Thorlabs, Newton, NJ). The electrolyte was vacuum-injected through a hole in the counter electrode, which was then sealed with a thermoplastic film and a glass coverslip.

### Solar Cell Characterization

Current–voltage measurements were performed in ambient air under AM 1.5G illumination by using a HelioSim-CL60 solar simulator (Voss Electronic GmbH). An X200 source meter (Ossila, Sheffield, UK) was used to assess the solar cell performance (scan speed 50 mV s^–1^). A circular mask was employed to confine the active solar cell area to 0.196 cm^2^.

### Electrochemical Impedance Spectroscopy

Electrochemical impedance spectra were recorded by using a PGSTAT12 potentiostat (Autolab) in the frequency range from 100 kHz to 0.1 Hz, modulating the voltage by 10 mV. The mathematical models for each circuit element are explained in eqs S1–S5 of the Supporting Information. The circuit models for each impedance analysis are indicated along each experiment and drawn in Figure S1.

To assess the TiO_2_ dense layers, an aqueous 0.01 M K_3_[Fe(CN)_6_], 0.01 M K_4_[Fe(CN)_6_], 0.1 M KCl solution was prepared, and the measurements were taken in an ambient atmosphere. A glassy carbon tip and a Pt wire were used as counter and reference electrode, respectively. The exposed area on the FTO|TiO_2_ glasses (working electrode) was masked to 1 cm diameter circles (0.785 cm^2^). The spectra were processed with an adapted version of the impedance fitting tool available on MATLAB file exchange.^24^ The blocking layer capacitance was further probed with electrolyte solutions containing (a) 0.02 M Cu(tmby)_2_TFSI and 0.004 M Cu(tmby)_2_TFSI, (b) 0.02 M Co(bpy)_3_(PF_6_)_2_, 0.005 M Co(bpy)_3_(PF_6_)_3_, and (c) 0.017 M 1,2-dimethyl-3-propylimidazolium iodide, 0.01 M lithium iodide, and 0.005 M iodine (all in 0.1 M acetonitrile solutions of tetrabutylammonium hexafluorophosphate).

Full DSC devices were illuminated with a white LED for impedance analysis and the response measured at different potentials. The total capacitance concerning electronic recombination was calculated by transferring each constant phase element to real capacitance according to eq 2, wherein *C*_el_ was calculated from *Q*_el_ and *R*_tr_, *C*_rec_ from *Q*_rec_ and *R*_rec_, and *C*_tot_ as the sum of the two.^25^

### Ellipsometry

For ellipsometry, the TiO_2_ layers were deposited onto SiO_*x*_|Si wafers (Inseto, Hampshire, UK) via spray pyrolysis as described above. Spectra were collected with a J.A. Woolean Inc. spectral ellipsometer (EC-400 M-2000F). The film thicknesses were fitted to the Ψ and Δ response between 250 and 1000 nm directly in the instrument software.

### Transient Photovoltage and Charge Collection Measurements

Electron recombination lifetimes were investigated by using a 1 W white LED (Luxeon Star). Kinetics in the solar cell were probed by applying square-wave modulations to the light intensity. The solar cell voltage response was tracked by a digital acquisition board (National Instruments) and fitted with first-order kinetic models.

The accumulated charge in the photoanodes of the DSC devices was measured by illuminating the cells at different light intensities at open circuit; then, the light was turned off, the potential simultaneously switched to short circuit and the current integrated over time. The collected charge was converted to charge density assuming a 6 mm diameter of the circular aperture, 4 μm anode thickness and 0.63 porosity.

## Results and Discussion

### Assessment of TiO_2_ Blocking Layers

**Figure 2:**
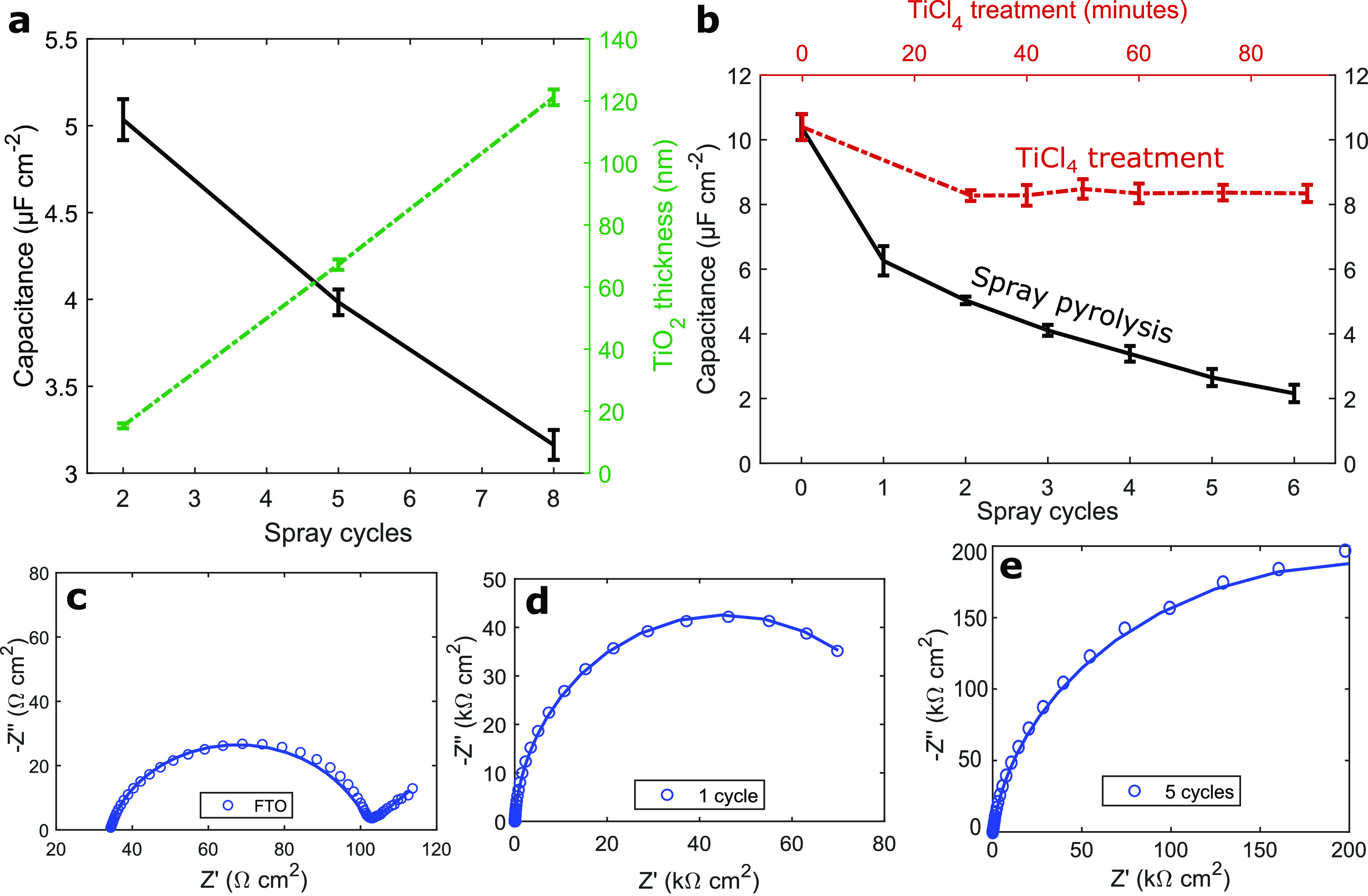
Characterization of TiO_2_ blocking layers with impedance spectroscopy. (a) Thickness of spray-pyrolyzed blocking layers and their Helmholtz capacitance. One spray iteration in our experiment corresponded to the deposition of 0.21 mL precursor per 100 cm^2^ substrate area. (b) Capacitance of spray-pyrolyzed (black) and TiCl_4_-bath-deposited (red) blocking layers. (c–e) Electrochemical impedance spectra of spray-pyrolyzed TiO_2_ blocking layers by thickness.

We describe a method of assessing TiO_2_ dense layers directly on conductive glass substrates. The blocking layers were deposited by repetitive spray pyrolysis of titanium bis(isopropoxide) bis(acetylacetonate).^26^ In our experiment, one spray iteration consumed 0.21 mL of precursor per 100 cm^2^ substrate area. For initial confirmation that the TiO_2_ thickness did increase with increased number of spray cycles, the height of the TiO_2_ films was assessed with ellipsometry; for reasons of reduced surface roughness, these measurements had to be performed on SiO_*x*_|Si wafers rather than FTO glass. The TiO_2_ thickness increased linearly and with little deviation with the number of spray repetitions (Figure 2a), allowing to use the latter as a measure for blocking layer thickness in all subsequent tests.

Masked areas (1 cm diameter circles) of blocking layers of different thickness (now on FTO substrates) were then immersed in an aqueous 0.01 M K_3_[Fe(CN)_6_], 0.01 M K_4_[Fe(CN)_6_], 0.1 M KCl solution (see scheme in Figure 1, middle), and the frequency domain response was measured (Figure 2a,b). In this and all subsequent graphs of electrochemical impedance spectra, the acquired data points are depicted as circles, and the fits to the corresponding equivalent circuit model as solid lines. Spectra of bare FTO were fitted to Randle’s equivalent circuit model (*R*([*RW*]*Q*), Figure S1-A1 and Figure 2c). For substrates comprising a TiO_2_ top layer, the (*RQ*) charge transfer time constant shifted to such low frequencies that the Warburg element was no longer visible and omitted from the circuit model (*R*(*RQ*), Figure S1-A2 and Figure 2d,e). The value of real capacitance *C* of the blocking layer was then converted from the pseudocapacitance *Q* of the constant phase element through a projection of the phase angle
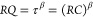
1and rearranging of the time constants τ as
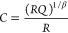
2where *R* is the resistance in parallel to the capacitor and β the exponent of the constant phase element.

The capacitance monotonically decreased with increasing spray repetitions, while the charge transfer resistance across the blocking layer *R*_CT_ increased. Examining the graph in Figure 2b (black line), it seems the capacitance drops with increasing distance, in line with the capacitor equation *C* = ϵ*A*/*d* (with area *A*, spacer dielectric constant ϵ, and distance *d*), implying the blocking layer can be seen as dielectric spacer between the electroactive FTO electrode and the [Fe(CN)_6_]^3–/4–^ redox electrolyte. We note here that this finding directly contrasts that of Fabregat-Santiago and co-workers, who found that the *R*_CT_ of their blocking layers even decreased with increased thickness;^21^ however, their deposition by sputtering seemed to give somewhat porous blocking layers, where an amplified deposition would indeed increase the active electrode/electrolyte interface and reduce *R*_CT_.

Spray-pyrolyzed TiO_2_ blocking layers are compared to the other widely known technique in the literature, namely chemical bath deposition from aqueous TiCl_4_ solution at elevated temperature (Figure 2b, red line).^27^ In contrast to deposition by spray pyrolysis, the capacitance of these blocking layers did not decrease after an initial drop. This is a direct confirmation of Liu et al.’s study, who demonstrated how chemical bath deposition, rather than coating a dense thin layer onto the electrode, leads to island-like crystal growth.^28^ In return, the area of exposed FTO substrate remains the dominating electrode/electrolyte interface. Their study highlighted the detrimental effect this has on recombination and therefore performance of the DSCs under ambient light, which is here underlined.

The aforementioned report of Fabregat-Santiago and co-workers directly used an organic I^–^/I_3_^–^ electrolyte to record the capacitance of their blocking layers.^21^ While we believe that the here-presented aqueous method with the [Fe(CN)_6_]^3–/4–^ couple is the more facile (and safe) experiment, we ran comparable experiments based on the Cu(tmby)_2_ redox couple, as will be employed in our DSCs below. The concentrations of active redox species Cu^I^(tmby)_2_TFSI and Cu^II^(tmby)_2_TFSI_2_ were kept at the same ratio but reduced 10-fold from the solar cell electrolyte concentrations to 0.02 and 0.004 M, respectively, to allow sufficient solubility of supporting electrolyte 0.1 M tetrabutylammonium hexafluorophosphate in acetonitrile. Generally, much higher resistances were observed for the nonaqueous redox electrolyte, readily for bare FTO electrodes (Figure S2). This is attributed to the increased polarizability of acetonitrile (ϵ_R_ = 33) as solvent medium over water (ϵ_R_ = 10). The Debye screening length λ_D_ in 1:1 electrolytes is
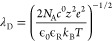
3with Avogadro’s number *N*_A_, bulk concentration *c*^0^, number of charges *z*, elementary charge *e*, vacuum and relative permittivity ϵ_0_/ϵ_R_, Boltzmann’s constant *k*_B_, and temperature *T*. In acetonitrile, the electric field from the electrode is present nearly twice as far (1.97 nm) compared to the aqueous solution (1.07 nm), leading to larger accumulations of static ions near the electrode and, in turn, higher charge transfer resistance.

An additional (RQ) element at high frequency appeared in the electrochemical impedance response of the electrodes in nonaqueous electrolytes (Figure S1-A3). For large blocking layer thicknesses, only the initial onset of the low-frequency semicircle was visible, potentially to be mistaken for a 45° line characteristic of ion diffusion (open-circuit Warburg, Figure S3); however, fitting equivalent circuit models showed a mismatched phase angle for ion diffusion and confirmed that this feature indeed was the onset of the increasingly large charge transfer element (*RQ*).

To keep all electrodes at equal potential and avoid polarizing interfaces, all above-discussed experiments were performed in a three-electrode setup without true reference electrode; instead, a glassy carbon tip and a Pt wire were used as counter and reference electrode, respectively. Assuming the density of states in the FTO substrate quasi-continuous and the TiO_2_ blocking layer as dielectric spacer, the capacitance of the Helmholtz double layer is independent of the redox potential of the electrolyte solution and therefore the electrode potential. This assumption holds as long as the electrochemical potential of the electrode (for I^–^/I_3_^–^, [Fe(CN)_6_]^3–/4–^, [Co(bpy)_3_]^3+/2+^, and [Cu(tmby)_2_]^2+/+^ 0.35, 0.370, 0.56, and 0.87 V vs SHE, respectively) does not approach either the conduction (−0.4 V) or valence band (2.8 V vs SHE) of TiO_2_. Still, we did investigate applying potentials across the blocking layer|electrolyte interface. In either direction of potential, *R*_CT_ increased (Figure S4), owing to solvated [Fe(CN)_6_]^3–/4–^ ions forming a Helmholtz electrochemical double layer at the electrode. Larger *R*_CT_ values, however, are generally unfavorable for the assessment of blocking layers as they constrain the measurement accuracy at low frequency. Interestingly, different behavior of *R*_CT_ depending on the applied potential was observed for the different electrochemical redox couples; we report these scans in Figures S4–S7 for completeness.

### Performance of TiO_2_ Blocking Layers by the Deposition Technique

**Figure 3:**
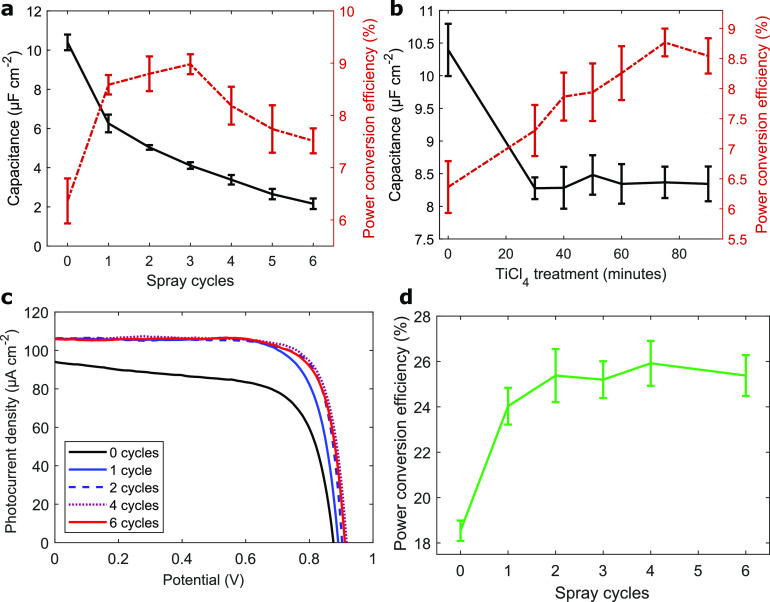
Relation of solar cell performance and blocking layer capacitance. (a) Blocking layers from spray pyrolysis (one spray iteration in our experiment corresponded to the deposition of 0.21 mL of precursor per 100 cm^2^ substrate area) and (b) from TiCl_4_ bath. (c) Current–voltage sweeps of Y123-DSCs under 1000 lx illumination from a fluorescent tube by blocking layer thickness. (d) Performance statistics.

To verify if the electrode capacitance can serve as calibration toward device performance, dye-sensitized solar cells were prepared based on either spray-pyrolized or TiCl_4_-bath-deposited TiO_2_ blocking layers. Evident from Figure 3a,b, clear maxima in power conversion efficiency are visible for blocking layers by either deposition method. For DSCs based on blocking layers from spray pyrolysis, exemplary *J*–*V* scans of cells of zero, three, and six spray repetitions are shown above in Figure 1c, and the importance of balancing *R*_series_ and *R*_shunt_ is pointed out (Figure 1d). The performance parameters of this cell series are summarized in Table S1. The best device at three spray repetitions attained 9.2% power conversion efficiency (average 8.98 ± 0.19%). Notably, this somewhat lower device performance compared to Saygili et al.’s 10.3% obtained with similar devices^29^ is attributed to deficiencies in the open-circuit voltage, owing to losses from additional recombination pathways aside from the TiO_2_ blocking layer.

The blocking layer capacitance assessed with aqueous [Fe(CN)_6_]^3–/4–^ appears an accurate measure to determine the correct thickness needed for high-performance DSCs with the Cu(tmby)_2_ electrolyte. Blocking layers by spray pyrolysis need to exhibit capacitances of 4.11 ± 0.17 μF cm^–2^ to perform best in DSC photoanodes; in fact, this value was confirmed from spray pyrolysis deposition performed by a different member of our laboratory. This confirms our proposition to use the aqueous capacitance as an accessible tool to verify the blocking layer quality. Still, it should be underlined that this value is not to serve as a global reference; indeed, in our experience, already a switch of the type or supplier of the FTO substrate potentially offsets the values of capacitance. In previous reports quantifying blocking layers in DSCs, Ito et al. and Yoo et al. increased the efficiency of their N719 I^–^/I_3_^–^ DSCs by up to 1% by optimization of spray pyrolysis and the TiCl_4_ precursor concentration, respectively.^12,14^ Goes et al. noticed an efficiency drop by 1.8% for similar systems at too thick blocking layer distances owing to increased series resistance.^21^ In our study, the efficiency gain from an optimized blocking layer thickness was more than 1.4-fold or an absolute 2.6% in power conversion efficiency under 1 sun. It seems therefore that the suppression of electronic recombination is of much greater importance for the use of high-performing metal redox shuttles such as [Cu(tmby)_2_]^2+/+^ compared to the kinetically hindered I^–^/I_3_^–^ two-electron redox couple.^30−32^

A maximum in power conversion efficiency was also observed for blocking layers by TiCl_4_ bath deposition (Figure 3b). At long times at elevated temperature, TiO_2_ aggregates grow in the bath solution and only subsequently attach to the electrodes. However, because of the above-discussed mechanics of the TiCl_4_ bath depositing islands rather than forming a thin film, this type of blocking layers does not seem suitable for capacitive assessment by [Fe(CN)_6_]^3–/4–^. Yella et al. had proposed that a better TiO_2_ dense layer could be obtained by raising the concentration of the TiCl_4_ solution up to 200 mM (from commonly 40–70 mM),^33^ which was last year verified by Kavan et al.^34^ This methodology may give blocking layers suitable for capacitance analysis; however, it has yet to see extensive tests in DSC devices.

Consequently, the devices were tested in ambient settings (Figure 3c and Table S2). The best cell (at *two* spray cycles) achieved 26.9% PCE. The performance of devices with no or too thin blocking layer is constrained by large shunt losses: Up to a third of the devices’ power output is compromised due to insufficient inhibition of recombination at the anode substrate, and the PCE dropped to just 18.%. In contrast to observations under sunlight, however, at (too) large blocking layer thicknesses, any performance drops due to series resistance are near-negligible: With current densities of only <150 μA cm^–2^ passing through the blocking layer, the voltage drop across the dense TiO_2_ is much less than under sunlight.

In most reports in the literature, device performances are higher for sprayed TiO_2_ blocking layers compared to their TiCl_4_-bath analogues; therefore, subsequent analysis focuses on the former.

### Correlation of Capacitance to Recombination

**Figure 4:**
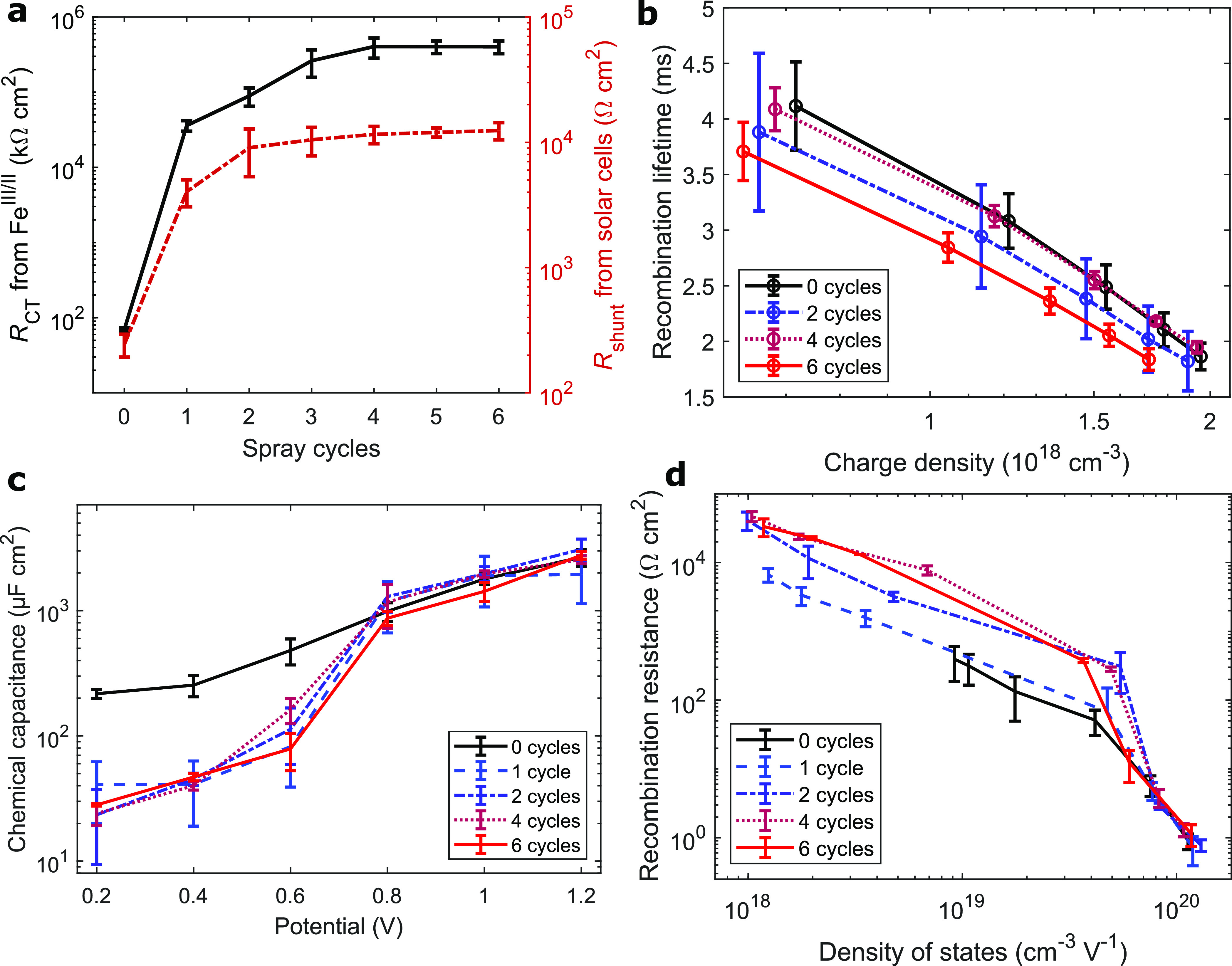
Electron transfers and recombination in DSCs by blocking layer thickness. (a) Charge transfer resistance from capacitance assessment in [Fe(CN)_6_]^3–/4–^ electrolyte and shunt resistance in DSCs with the [Cu(tmby)_2_]^2+/+^ redox shuttle. One spray iteration in our experiment corresponded to the deposition of 0.21 mL of precursor per 100 cm^2^ substrate area. (b) Electron recombination lifetimes (around *V*_OC_). (c) Chemical capacitance of the FTO|TiO_2_|dye|electrolyte interface versus potential by blocking layer thickness. (d) Electronic recombination resistance versus density of electronic states; horizontal errorbars are omitted for visibility here, shown in Figure S9.

The charge transfer resistance *R*_CT_ with the aqueous [Fe(CN)_6_]^3–/4–^ couple directly relates to the *R*_shunt_ from *J*–*V* sweeps of the DSCs with the [Cu(tmby)_2_]^2+/+^ redox shuttle: Both parameters increase with repetitions of spray cycles (Figure 4a). At very large blocking layer thicknesses, this effect even becomes visible in the devices’ series resistance (Figure 1d and Figure S10a). Notably, an exemption to this trend is the series resistance in the absence of any TiO_2_ dense layer.

In more in-depth analysis, the electronic recombination lifetimes in DSC devices were examined by transient photovoltage analysis (Figure 3b). At higher light intensities, and thus larger charge density in the (mesoporous) TiO_2_, the recombination lifetime of electrons decreases, independent of blocking layer thickness. As expected, no significant differences by blocking layer thickness in this measurement demonstrate that the recombination in these settings (around *V*_OC_) dominantly occurs across the mesoporous-TiO_2_|dye|electrolyte interface.

To target an examination of the recombination at the FTO|blocking-TiO_2_|electrolyte interface, electrochemical impedance spectra of full DSC devices under illumination were recorded (Figure S8). At high potentials, the spectra were fitted to Bisquert’s transmission-recombination line (Figure S1-B1);^35^ toward *J*_SC_, the charge transfer resistance *R*_CT_ outgrew contributions of both transmission line *R*_tr_ and the Warburg diffusion response *Z*_W_, so that a simplified equivalent circuit model was applied (Figure S1-B2), in accordance with the literature.^36,37^ The chemical capacitance of the devices can be interpreted as electrons in the photoanode in vicinity of the electrolyte and, as such, at risk of recombining with the oxidized redox species in the electrolyte solution. At high voltages, no differences in chemical capacitance are visible depending on the blocking layer thickness (Figure 4c). This is in accordance with expectation and observations from transient photovoltage tests in Figure 4b, as the vast majority of electrons populate the mesoporous TiO_2_ under these conditions, and the TiO_2_ blocking layer plays only a minor role. Contrarily, when the potential across the DSCs was lowered and the TiO_2_ conduction band was depopulated, the chemical capacitance dropped sharply in all devices except those lacking any TiO_2_ blocking layer. At low potentials, this capacitance now directly corresponds to the FTO|blocking-TiO_2_|electrolyte interface, and it is possible to estimate the amount of charge directly exposed to the electrolyte at the anode substrate, which remained around 1 order of magnitude increased in devices lacking a TiO_2_ blocking layer (Figure 4c). This gives evidence that electrons in the FTO substrate are not sufficiently shielded from the redox electrolyte.

The density of states in a DSC photoanode *D* at potential *E* is calculated from the capacitance *C* of the photoanode as
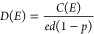
4as proposed by Durrant and co-workers,^38^ with the elementary charge *e*, the thickness of the TiO_2_ film *d* (here 4 μm), and the porosity *p* (assumed 0.63 after Wang et al.^39^). Visible in Figure 4d (graph magnified with horizontal errorbars in Figure S9) is the growing recombination resistance with decreasing density of electronic states in vicinity of the redox electrolyte. At low potentials (and therefore low charge density), the recombination resistance rises to similar values for all cells based on two or more spray pyrolysis cycles. The mesoporous TiO_2_ is depopulated and nearly insulating; as such, this recombination resistance at low potentials translates to charge transfer across the FTO|blocking-TiO_2_|electrolyte interface. Notably, in devices based on the thinnest blocking layer of just one spray pyrolysis cycle, a similar conduction band depopulation was reached, yet the recombination resistance was still somewhat lower. In the absence of any blocking layer, the density of electronic states does not drop below 10^19^ cm^–3^ V^–1^, and the recombination resistance does not exceed hundreds of ohms, as such not sufficient to effectively shield electron recombination in DSCs. The recombination resistance toward short-circuit (the leftmost points) now directly correlates to the shunt resistance obtained from *J*–*V* curves (Figure S10b). Comparing Figures 4d and 3a, the conclusion is reached that the ideal blocking layer thickness is reached when the recombination resistance at low charge density converges.

Electrochemical impedance spectra of the DSC devices were recorded in the dark (Figure S10c,d). While similar resistances for charge transfer across the meso-TiO_2_|dye|electrolyte interface of tens of ohms are observed at high voltages, the mesoporous TiO_2_ rapidly becomes insulating as it is depopulated and no photoexcited electrons are injected. Therefore, the resistance grows above 1 MΩ within 0.4 V. Similar to observations under sunlight, in the absence of any blocking layer, the FTO is directly involved in the electrochemical cell. The observed density of states remains high, and the “recombination” resistance is an order of magnitude higher. The charge transfer time constant τ = *R*_rec_*C*_rec_ = (*R*_rec_*Q*_rec_)^β^ decreases with increasing charge in the TiO_2_ (Figure 3c). Notably, at high charge densities, the charge transfer approaches the lifetime constant observed from photocurrent transients.

## Conclusion and Outlook

The electrochemical properties of TiO_2_ blocking layers were investigated in aqueous [Fe(CN)_6_]^3–/4–^ solution. Their capacitance dropped monotonically with the repetitions of spray pyrolysis, indicating electronic protection of the FTO electrode. In return, a desired capacitance of 4.1 μF cm^–2^ was determined to be required for optimal performance (9.2%) of photoanodes in DSC devices based on the Cu(tmby)_2_ electrolyte. Deposition by TiCl_4_ treatment did not provide sufficient electronic shielding. The effects of the blocking layer on electronic recombination in DSCs were probed with transient photovoltage methods and electrochemical impedance analysis: To provide sufficient shielding of the FTO anode, the blocking layer is critical and lowers the chemical capacitance of the photoanode below 100 μF cm^–2^ at potentials toward *J*_SC_. If this criterion is met, the recombination resistance increases over 1 kΩ and devices exhibit the highest performance.

We have here demonstrated a feasible and facile method to assess TiO_2_ blocking layers at an early stage during the fabrication of dye-sensitized solar cells. This methodology will allow for the direct evaluation of carrier-selective dense layers, ranging from DSC blocking layers to their analogues in other thin-film technologies.

## Supporting Information Available

Equivalent circuit models and the mathematical derivation of their elements; impedance analysis of blocking layers in various electrolytes and versus potential; photovoltaic parameters of DSC devices; impedance analysis of full DSC devices; electronic recombination versus density of states with errorbars (PDF)

## References

[ref1] Kakiage K.; Aoyama Y.; Yano T.; Oya K.; Fujisawa J.-i.; Hanaya M. Highly-efficient dye-sensitized solar cells with collaborative sensitization by silyl-anchor and carboxy-anchor dyes. Chem. Commun. 2015, 51, 15894–15897. 10.1039/C5CC06759F. 26393334

[ref2] Ji J.-M.; Zhou H.; Eom Y. K.; Kim C. H.; Kim H. K. 14.2% Efficiency Dye-Sensitized Solar Cells by Co-sensitizing Novel Thieno[3,2-b]indole-Based Organic Dyes with a Promising Porphyrin Sensitizer. Adv. Energy Mater. 2020, 10, 2000124 10.1002/aenm.202000124.

[ref3] Huaulmé Q.; Mwalukuku V. M.; Joly D.; Liotier J.; Kervella Y.; Maldivi P.; Narbey S.; Oswald F.; Riquelme A. J.; Anta J. A.; Demadrille R. Photochromic dye-sensitized solar cells with light-driven adjustable optical transmission and power conversion efficiency. Nature Energy 2020, 5, 468–477. 10.1038/s41560-020-0624-7. 35475116 PMC7612663

[ref4] Gerischer H. The impact of semiconductors on the concepts of electrochemistry. Electrochim. Acta 1990, 35, 1677–1699. 10.1016/0013-4686(90)87067-C.

[ref5] O’Regan B.; Grätzel M. A low-cost, high-efficiency solar cell based on dye-sensitized colloidal TiO_2_ films. Nature 1991, 353, 737–740. 10.1038/353737a0.

[ref6] Hagfeldt A.; Boschloo G.; Sun L.; Kloo L.; Pettersson H. Dye-Sensitized Solar Cells. Chem. Rev. 2010, 110, 6595–6663. 10.1021/cr900356p. 20831177

[ref7] Muñoz-García A. B.; Benesperi I.; Boschloo G.; Concepcion J. J.; Delcamp J. H.; Gibson E. A.; Meyer G. J.; Pavone M.; Pettersson H.; Hagfeldt A.; Freitag M. Dye-sensitized solar cells strike back. Chem. Soc. Rev. 2021, 50, 12450–12550. 10.1039/D0CS01336F. 34590638 PMC8591630

[ref8] Benesperi I.; Michaels H.; Freitag M. The researcher’s guide to solid-state dye-sensitized solar cells. J. Mater. Chem. C 2018, 6, 11903–11942. 10.1039/C8TC03542C.

[ref9] Freitag M.; Teuscher J.; Saygili Y.; Zhang X.; Giordano F.; Liska P.; Hua J.; Zakeeruddin S. M.; Moser J.-E.; Grätzel M.; Hagfeldt A. Dye-sensitized solar cells for efficient power generation under ambient lighting. Nat. Photonics 2017, 11, 372–378. 10.1038/nphoton.2017.60.

[ref10] Michaels H.; Rinderle M.; Freitag R.; Benesperi I.; Edvinsson T.; Socher R.; Gagliardi A.; Freitag M. Dye-sensitized solar cells under ambient light powering machine learning: towards autonomous smart sensors for the internet of things. Chem. Sci. 2020, 11, 2895–2906. 10.1039/C9SC06145B. 34122790 PMC8157489

[ref11] Zhang D.; Stojanovic M.; Ren Y.; Cao Y.; Eickemeyer F. T.; Socie E.; Vlachopoulos N.; Moser J.-E.; Zakeeruddin S. M.; Hagfeldt A.; Grätzel M. A molecular photosensitizer achieves a Voc of 1.24V enabling highly efficient and stable dye-sensitized solar cells with copper(II/I)-based electrolyte. Nat. Commun. 2021, 12, 1777 10.1038/s41467-021-21945-3. 33741953 PMC7979847

[ref12] Ito S.; Liska P.; Comte P.; Charvet R.; Péchy P.; Bach U.; Schmidt-Mende L.; Zakeeruddin S. M.; Kay A.; Nazeeruddin M. K.; Grätzel M. Control of dark current in photoelectrochemical (TiO_2_/I–I3-) and dye-sensitized solar cells. Chem. Commun. 2005, 4351–4353. 10.1039/b505718c. 16113745

[ref13] Waita S. M.; Aduda B. O.; Mwabora J. M.; Niklasson G. A.; Granqvist C. G.; Boschloo G. Electrochemical characterization of TiO_2_ blocking layers prepared by reactive DC magnetron sputtering. J. Electroanal. Chem. 2009, 637, 79–83. 10.1016/j.jelechem.2009.10.004.

[ref14] Yoo B.; Kim K.; Bang S.; Ko M.; Kim K.; Park N. Chemically deposited blocking layers on FTO substrates: Effect of precursor concentration on photovoltaic performance of dye-sensitized solar cells. J. Electroanal. Chem. 2010, 638, 161–166. 10.1016/j.jelechem.2009.10.005.

[ref15] Unger E. L.; Spadavecchia F.; Nonomura K.; Palmgren P.; Cappelletti G.; Hagfeldt A.; Johansson E. M. J.; Boschloo G. Effect of the Preparation Procedure on the Morphology of Thin TiO_2_ Films and Their Device Performance in Small-Molecule Bilayer Hybrid Solar Cells. ACS Appl. Mater. Interfaces 2012, 4, 5997–6004. 10.1021/am301604x. 23066994

[ref16] Boschloo G. K.; Goossens A.; Schoonman J. Photoelectrochemical Study of Thin Anatase TiO_2_ Films Prepared by Metallorganic Chemical Vapor Deposition. J. Electrochem. Soc. 1997, 144, 1311–1317. 10.1149/1.1837590.

[ref17] Cameron P. J.; Peter L. M.; Hore S. How Important is the Back Reaction of Electrons via the Substrate in Dye-Sensitized Nanocrystalline Solar Cells?. J. Phys. Chem. B 2005, 109, 930–936. 10.1021/jp0405759. 16866461

[ref18] Zhang S.-T.; Roussel H.; Chaix-Pluchery O.; Langlet M.; Muñoz-Rojas D.; Bellet D.; Klein A. Polymorphism of the Blocking TiO_2_ Layer Deposited on F:SnO_2_ and Its Influence on the Interfacial Energetic Alignment. J. Phys. Chem. C 2017, 121, 17305–17313. 10.1021/acs.jpcc.7b04893.

[ref19] Kavan L.; Grätzel M. Highly efficient semiconducting TiO_2_ photoelectrodes prepared by aerosol pyrolysis. Electrochim. Acta 1995, 40, 643–652. 10.1016/0013-4686(95)90400-W.

[ref20] Cameron P. J.; Peter L. M. Characterization of Titanium Dioxide Blocking Layers in Dye-Sensitized Nanocrystalline Solar Cells. J. Phys. Chem. B 2003, 107, 14394–14400. 10.1021/jp030790+.

[ref21] Góes M. S.; Joanni E.; Muniz E. C.; Savu R.; Habeck T. R.; Bueno P. R.; Fabregat-Santiago F. Impedance Spectroscopy Analysis of the Effect of TiO_2_ Blocking Layers on the Efficiency of Dye Sensitized Solar Cells. J. Phys. Chem. C 2012, 116, 12415–12421. 10.1021/jp301694r.

[ref22] Kavan L.; Tétreault N.; Moehl T.; Grätzel M. Electrochemical Characterization of TiO_2_ Blocking Layers for Dye-Sensitized Solar Cells. J. Phys. Chem. C 2014, 118, 16408–16418. 10.1021/jp4103614.

[ref23] Ellis H.; Vlachopoulos N.; Häggman L.; Perruchot C.; Jouini M.; Boschloo G.; Hagfeldt A. PEDOT Counter Electrodes for Dye-Sensitized Solar Cells Prepared by Aqueous Micellar Electrodeposition. Electrochim. Acta 2013, 107, 45–51. 10.1016/j.electacta.2013.06.005.

[ref24] Dellis J.-L. MATLAB Zfit curve fitting tool. https://se.mathworks.com/matlabcentral/fileexchange/19460-zfit, acessed 2018-10-18.

[ref25] Sarker S.; Ahammad A. J. S.; Seo H. W.; Kim D. M. Electrochemical Impedance Spectra of Dye-Sensitized Solar Cells: Fundamentals and Spreadsheet Calculation. International Journal of Photoenergy 2014, 2014, 1 10.1155/2014/851705.

[ref26] Peng B.; Jungmann G.; Jäger C.; Haarer D.; Schmidt H.-W.; Thelakkat M. Systematic investigation of the role of compact TiO_2_ layer in solid state dye-sensitized TiO_2_ solar cells. Coord. Chem. Rev. 2004, 248, 1479–1489. 10.1016/j.ccr.2004.02.008.

[ref27] Choi H.; Nahm C.; Kim J.; Moon J.; Nam S.; Jung D.-R.; Park B. The effect of TiCl4-treated TiO_2_ compact layer on the performance of dye-sensitized solar cell. Curr. Appl. Phys. 2012, 12, 737–741. 10.1016/j.cap.2011.10.011.

[ref28] Liu I.-P.; Lin W.-H.; Tseng-Shan C.-M.; Lee Y.-L. Importance of Compact Blocking Layers to the Performance of Dye-Sensitized Solar Cells under Ambient Light Conditions. ACS Appl. Mater. Interfaces 2018, 10, 38900–38905. 10.1021/acsami.8b13181. 30338984

[ref29] Saygili Y.; Söderberg M.; Pellet N.; Giordano F.; Cao Y.; Muñoz-García A. B.; Zakeeruddin S. M.; Vlachopoulos N.; Pavone M.; Boschloo G.; Kavan L.; Moser J.-E.; Grätzel M.; Hagfeldt A.; Freitag M. Copper Bipyridyl Redox Mediators for Dye-Sensitized Solar Cells with High Photovoltage. J. Am. Chem. Soc. 2016, 138, 15087–15096. 10.1021/jacs.6b10721. 27749064

[ref30] Michaels H.; Benesperi I.; Edvinsson T.; Muñoz-Garcia A. B.; Pavone M.; Boschloo G.; Freitag M. Copper Complexes with Tetradentate Ligands for Enhanced Charge Transport in Dye-Sensitized Solar Cells. Inorganics 2018, 6, 53 10.3390/inorganics6020053.

[ref31] Saygili Y.; Stojanovic M.; Michaels H.; Tiepelt J.; Teuscher J.; Massaro A.; Pavone M.; Giordano F.; Zakeeruddin S. M.; Boschloo G.; Moser J.-E.; Grätzel M.; Muñoz-García A. B.; Hagfeldt A.; Freitag M. Effect of Coordination Sphere Geometry of Copper Redox Mediators on Regeneration and Recombination Behavior in Dye-Sensitized Solar Cell Applications. ACS Applied Energy Materials 2018, 1, 4950–4962. 10.1021/acsaem.8b00957.

[ref32] Benesperi I.; Michaels H.; Edvinsson T.; Pavone M.; Probert M. R.; Waddell P.; Muñoz-García A. B.; Freitag M. Dynamic dimer copper coordination redox shuttles. Chem. 2021, 10.1016/j.chempr.2021.10.017.

[ref33] Yella A.; Heiniger L.-P.; Gao P.; Nazeeruddin M. K.; Grätzel M. Nanocrystalline Rutile Electron Extraction Layer Enables Low-Temperature Solution Processed Perovskite Photovoltaics with 13.7% Efficiency. Nano Lett. 2014, 14, 2591–2596. 10.1021/nl500399m. 24628563

[ref34] Kavan L.; Vlckova Zivcova Z.; Zlamalova M.; Zakeeruddin S. M.; Grätzel M. Electron-Selective Layers for Dye-Sensitized Solar Cells Based on TiO_2_ and SnO_2_. J. Phys. Chem. C 2020, 124, 6512–6521. 10.1021/acs.jpcc.9b11883.

[ref35] Bisquert J.; Grätzel M.; Wang Q.; Fabregat-Santiago F. Three-Channel Transmission Line Impedance Model for Mesoscopic Oxide Electrodes Functionalized with a Conductive Coating. J. Phys. Chem. B 2006, 110, 11284–11290. 10.1021/jp0611727. 16771398

[ref36] Sol. Energy Mater. Sol. Cells 2005, 87, 117–131.

[ref37] Fabregat-Santiago F.; Garcia-Belmonte G.; Mora-Seró I.; Bisquert J. Characterization of nanostructured hybrid and organic solar cells by impedance spectroscopy. Phys. Chem. Chem. Phys. 2011, 13, 9083–9118. 10.1039/c0cp02249g. 21468446

[ref38] O’Regan B. C.; Bakker K.; Kroeze J.; Smit H.; Sommeling P.; Durrant J. R. Measuring Charge Transport from Transient Photovoltage Rise Times. A New Tool To Investigate Electron Transport in Nanoparticle Films. J. Phys. Chem. B 2006, 110, 17155–17160. 10.1021/jp062761f. 16928011

[ref39] Wang Q.; Moser J.-E.; Grätzel M. Electrochemical Impedance Spectroscopic Analysis of Dye-Sensitized Solar Cells. J. Phys. Chem. B 2005, 109, 14945–14953. 10.1021/jp052768h. 16852893

