# Supplementary material for: Assessment of TiO2 Blocking Layers for CuII/I-Electrolyte Dye-Sensitized Solar Cells by Electrochemical Impedance Spectroscopy
Source: ACS Appl Energy Mater. 2022 Feb 11;5(2):1933–41. doi: 10.1021/acsaem.1c03433 (PMC9096799; doi:10.1021/acsaem.1c03433)
Supplement: Supplementary file 1 — ae1c03433_si_001.pdf [file ae1c03433_si_001.pdf]

Supporting Information: Assessment of TiO_2 blocking layers for $Cu^{II/I}$-electrolyte dye-sensitized solar cells by electrochemical impedance spectroscopy

Hannes Michaels[†,‡] and Marina Freitag[*,‡,†]

†*Department of Chemistry - Ångström Laboratory, Uppsala University, Uppsala, Sweden*

‡*School of Natural and Environmental Science, Newcastle University, Newcastle, UK*

E-mail: marina.freitag@newcastle.ac.uk

Equivalent circuits

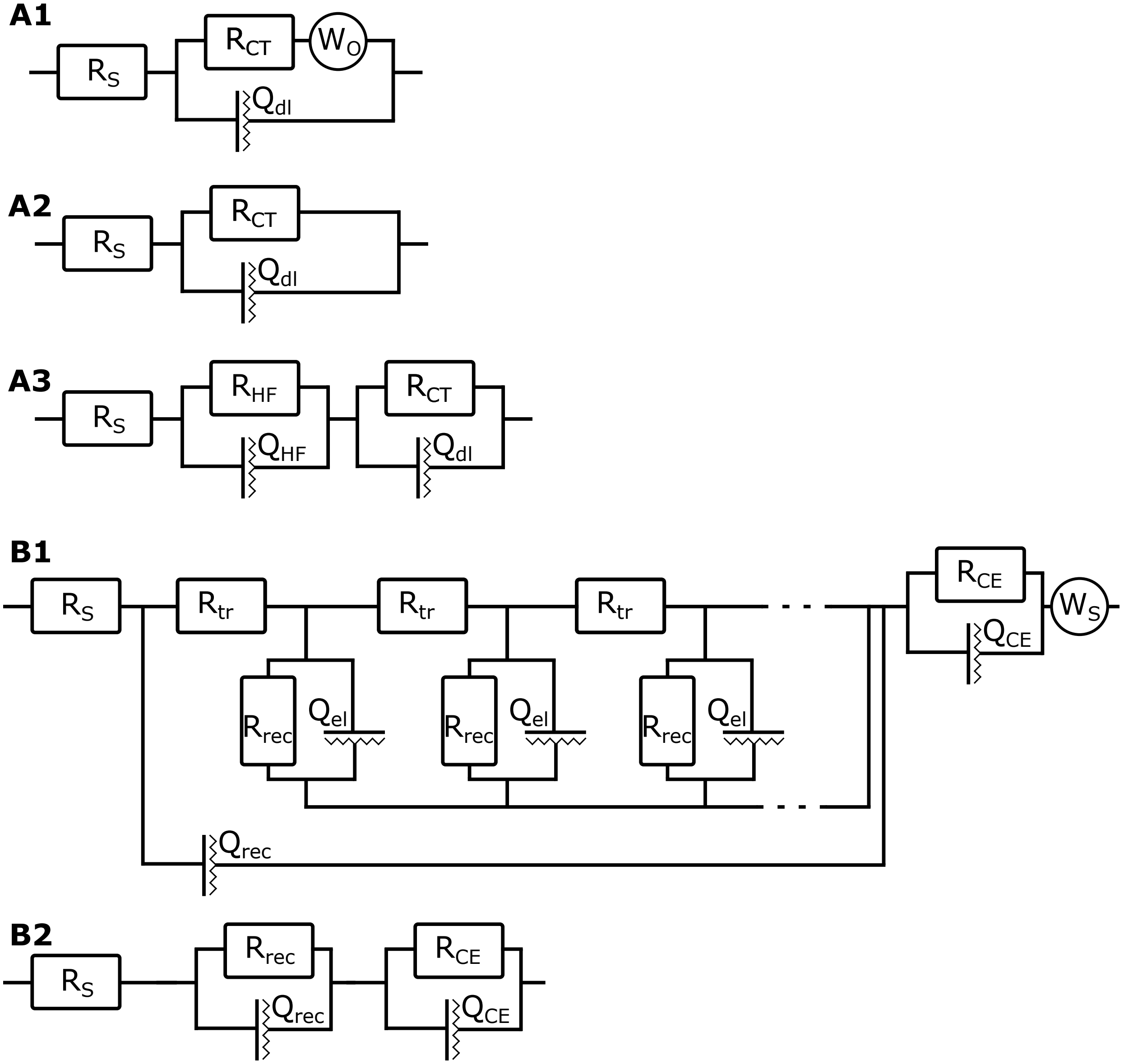

Figure S1: Equivalent circuit models used for impedance analysis in this study. **A1-2**: R_S, series resistance; R_{CT}, charge transfer resistance across the FTO|blocking-TiO_2|$[Fe(CN)_6]^{3-/4-}$ interface; Q_{dl}, constant phase element of the electrochemical double layer; W_O, open-circuit Warburg element; **A3**: R/Q_{HF}, additional RQ element at high frequency for blocking layer tests in non-aqueous electrolytes; **B1-2**: R_{tr}, transport resistance through the mesoporous TiO_2; Q_{el}, pseudo-capacitance from electronic accumulation in the photoanode; R/Q_{rec}, recombination resistance and constant-phase element pseudo-capacitance across the TiO_2|dye|$[Cu(tmby)_2]^{2+/+}$ interface; R/Q_{CE}, charge transfer resistance and pseudo-capacitance at the counter electrode; W_O, short-circuit Warburg element.

Equivalent circuit elements

The response of a constant phase element Q, as a function of frequency ω, is

$$Z_Q(\omega) = \frac{1}{Q(i\omega)^\beta} \tag{S1}$$

The open-circuit Warburg element follows

$$Z_{\mathrm{Wo}}(\omega) = \frac{R_\mathrm{W}}{\sqrt{T_\mathrm{W} i\omega}} \coth(\sqrt{T_\mathrm{W} i\omega}) \tag{S2}$$

The short-circuit Warburg element follows

$$Z_{\mathrm{Ws}}(\omega) = \frac{R_\mathrm{W}}{\sqrt{T_\mathrm{W} i\omega}} \tanh(\sqrt{T_\mathrm{W} i\omega}) \tag{S3}$$

In either case, the Warburg resistance R_W is

$$R_\mathrm{W} = \frac{RT\delta}{\sqrt{2} z^2 F^2 A} \left(\frac{1}{c_O D_O} + \frac{1}{c_R D_R} \right) \tag{S4}$$

and the corresponding time constant T_W

$$T_\mathrm{W} = \frac{\delta^2}{D} \tag{S5}$$

with the gas constant R, temperature T, electrode distance δ, charge z, Faraday constant F, electrode area A and the concentration c and diffusion coefficient D of the oxidized O and reduced electrolyte species R, respectively. We note here that Equation SS4 is shown in its general form; in a common assumption for DSC electrolytes, the diffusion is limited by the oxidized mediator species only due to the far lower concentration, the terms in the parenthesis simplify to $1/(c_O D_O)$.

Probing blocking layers with impedance analysis

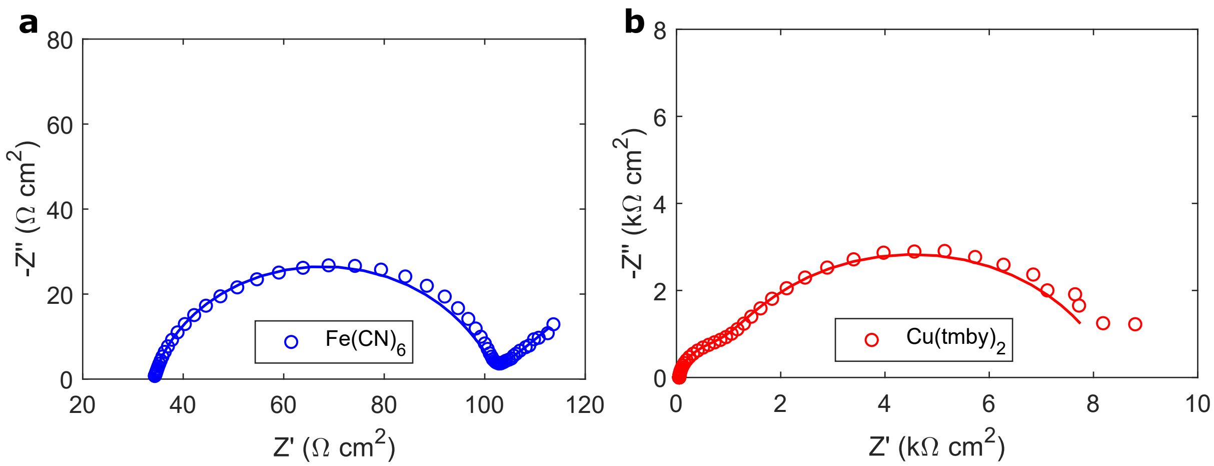

Figure S2: Electrochemical impedance spectra of bare FTO electrodes in **a**, aqueous 0.01 M $K_3[Fe(CN)_6]$, 0.01 M $K_4[Fe(CN)_6]$, 0.1 M KCl and **b**, 0.02 M $Cu(tmby)_2$TFSI, 0.004 M $Cu(tmby)_2$TFSI in 0.1 M acetonitrile solution of tetrabutylammonium hexafluorophosphate.

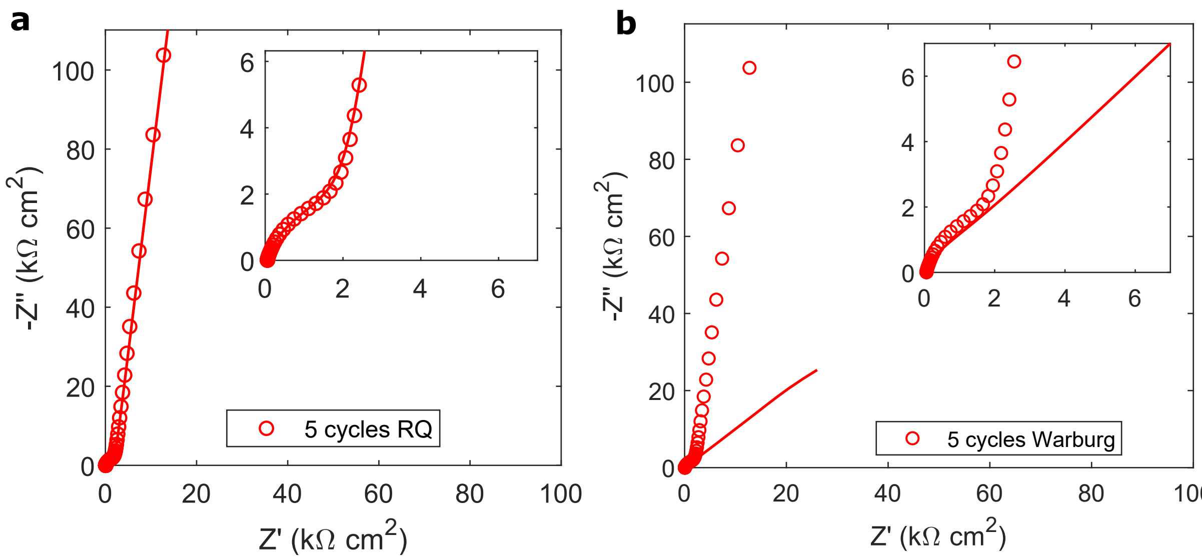

Figure S3: Electrochemical impedance spectrum of a comparably thick blocking layer (here from five cycles of spray pyrolysis), in 0.02 M Cu(tmby)$_2$TFSI, 0.004 M Cu(tmby)$_2$TFSI in 0.1 M acetonitrile solution of tetrabutylammonium hexafluorophosphate. **a**, applying a R[(RQ)(RQ)] circuit model (see Fig. S1 A3) and **b**, incorrectly applying a R([RW]Q) diffusion model (Fig. S1 A1).

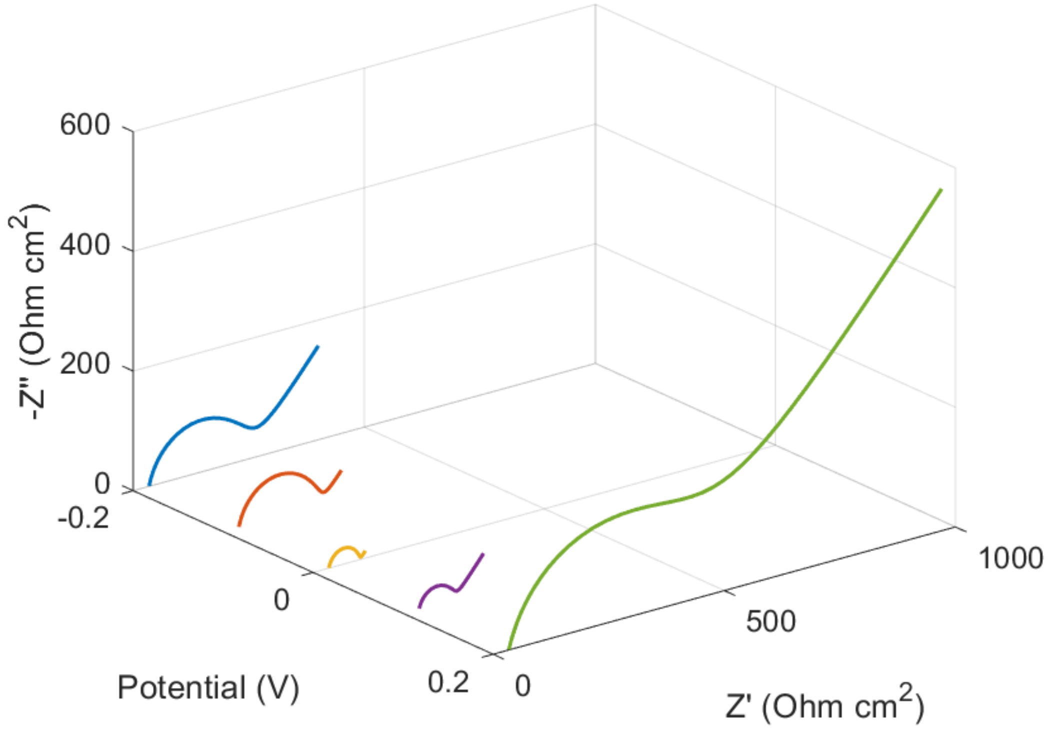

Figure S4: Electrochemical impedance spectra of a bare FTO electrode in aqueous 0.01 M $K_3[Fe(CN)_6]$, 0.01 M $K_4[Fe(CN)_6]$, 0.1 M KCl, versus potential.

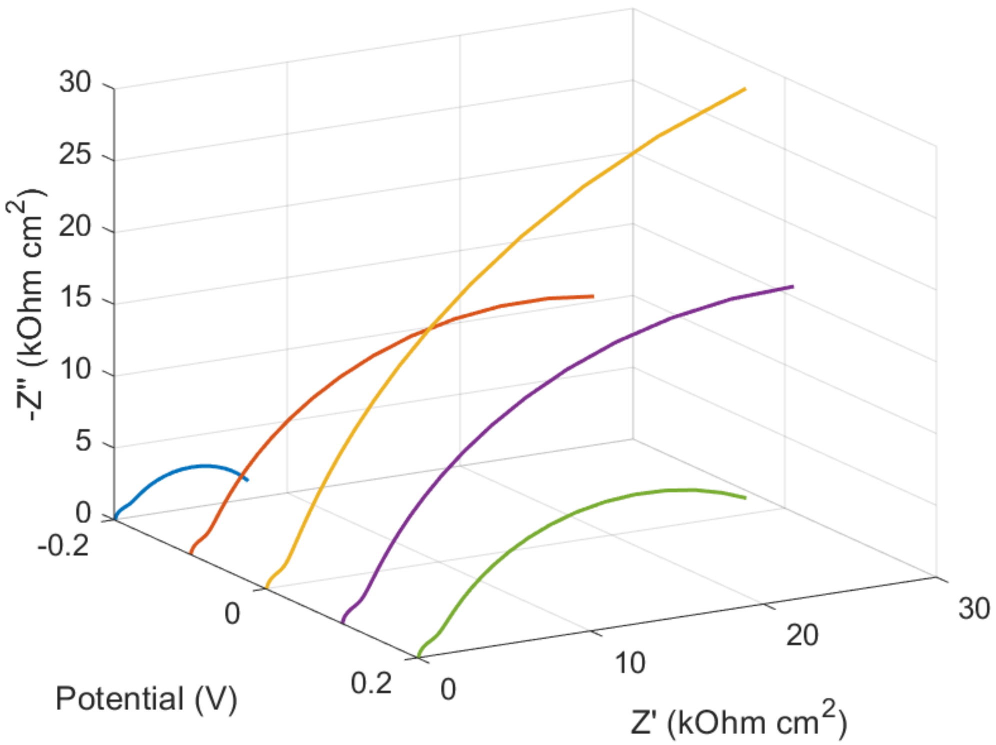

Figure S5: Electrochemical impedance spectra of a bare FTO electrode in 0.02 M $Cu(tmby)_2TFSI$, 0.004 M $Cu(tmby)_2TFSI$ in 0.1 M acetonitrile solution of tetrabutylammonium hexafluorophosphate, versus potential.

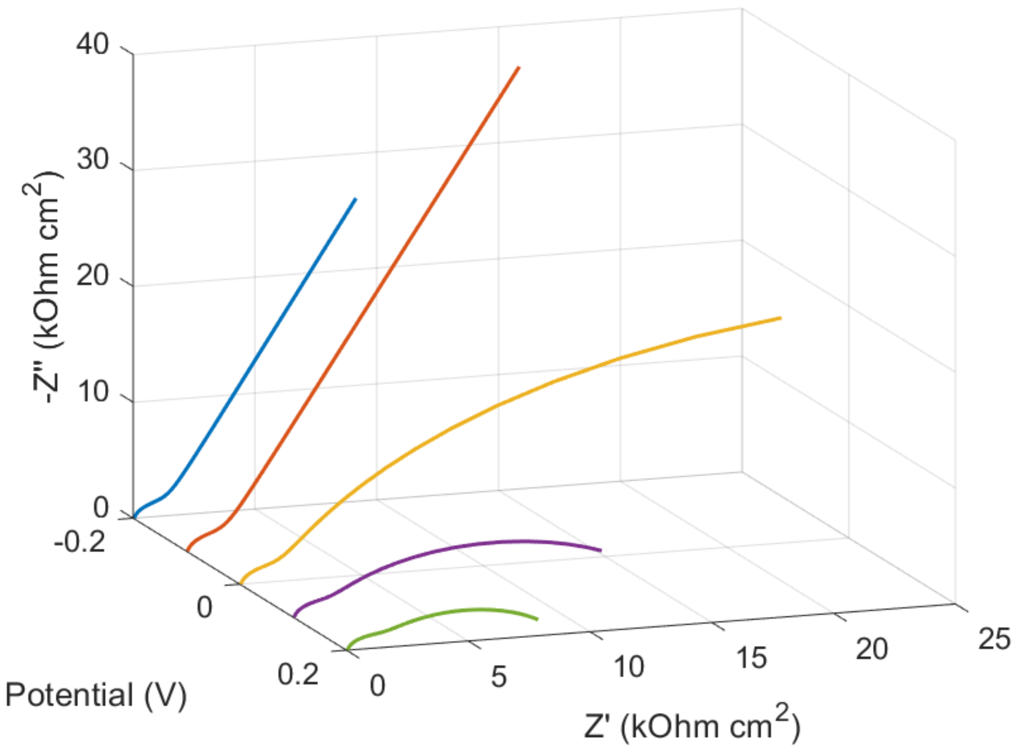

Figure S6: Electrochemical impedance spectra of a bare FTO electrode in 0.02 M $Co(bpy)_3(PF_6)_2$, 0.005 M $Co(bpy)_3(PF_6)_3$ in 0.1 M acetonitrile solution of tetrabutylammonium hexafluorophosphate, versus potential.

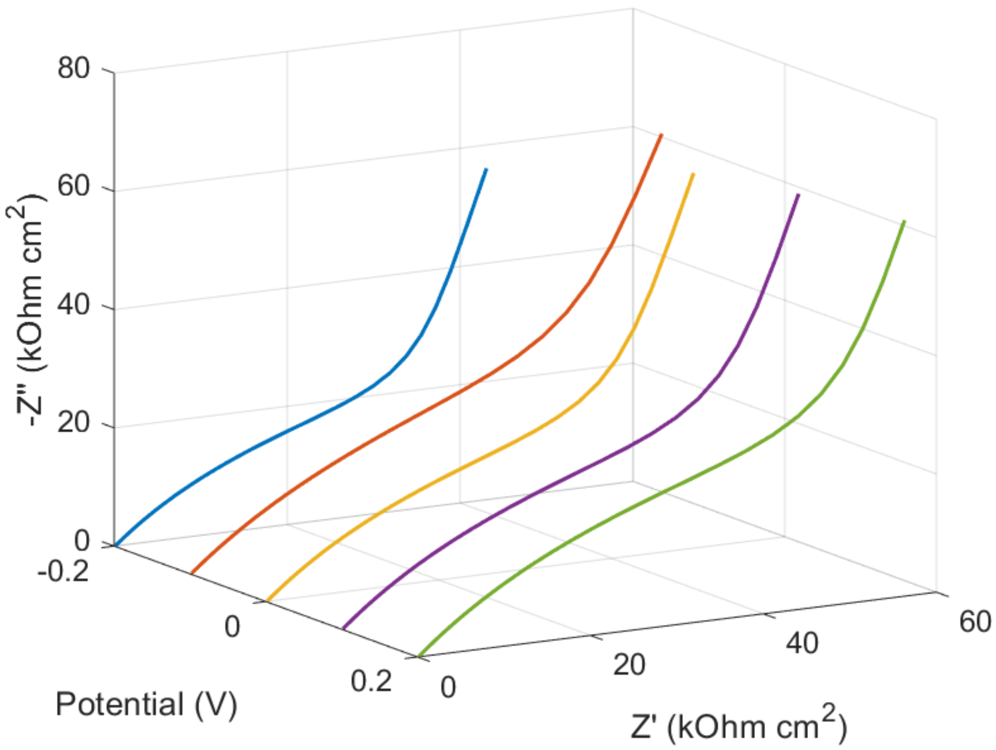

Figure S7: Electrochemical impedance spectra of a bare FTO electrode in 0.017 M 1,2-dimethyl-3-propylimidazolium iodide, 0.01 M lithium iodide and 0.005 M iodine in 0.1 M acetonitrile solution of tetrabutylammonium hexafluorophosphate, versus potential.

Photovoltaic cells by blocking layer thickness

Table S1: Photovoltaic parameters corresponding to the current-voltage sweeps in Fig. 1 and statistical analysis in Fig. 3a.

Spray cycles	V_{OC} (V)	J_{SC} (mA cm^{-2})	FF	PCE (%)
0	0.928±0.03	11.3±0.53	0.613±0.05	6.36±0.43
1	0.969±0.03	11.8±0.35	0.760±0.01	8.58±0.19
2	0.980±0.02	11.8±0.13	0.764±0.02	8.79±0.33
3	0.978±0.01	12.1±0.20	0.771±0.02	8.98±0.19
4	0.980±0.02	11.3±0.35	0.743±0.01	8.19±0.36
5	0.976±0.03	11.4±0.35	0.698±0.01	7.74±0.45
6	0.982±0.01	11.1±0.16	0.687±0.03	7.51±0.24

Table S2: Photovoltaic parameters under ambient illumination corresponding to Fig. 3c-d.

Spray cycles	V_{OC} (V)	J_{SC} (µA cm^{-2})	FF	PCE (%)
0	0.872±0.02	92±2	0.685±0.03	18.5±0.5
1	0.895±0.01	106±2	0.779±0.02	24.9±0.9
2	0.917±0.02	106±2	0.795±0.04	25.4±1.3
3	0.914±0.02	106±2	0.783±0.02	25.2±0.5
4	0.920±0.02	106±2	0.806±0.02	25.9±1.1
6	0.914±0.03	106±2	0.791±0.01	25.5±1.3

Impedance analysis of full DSC devices

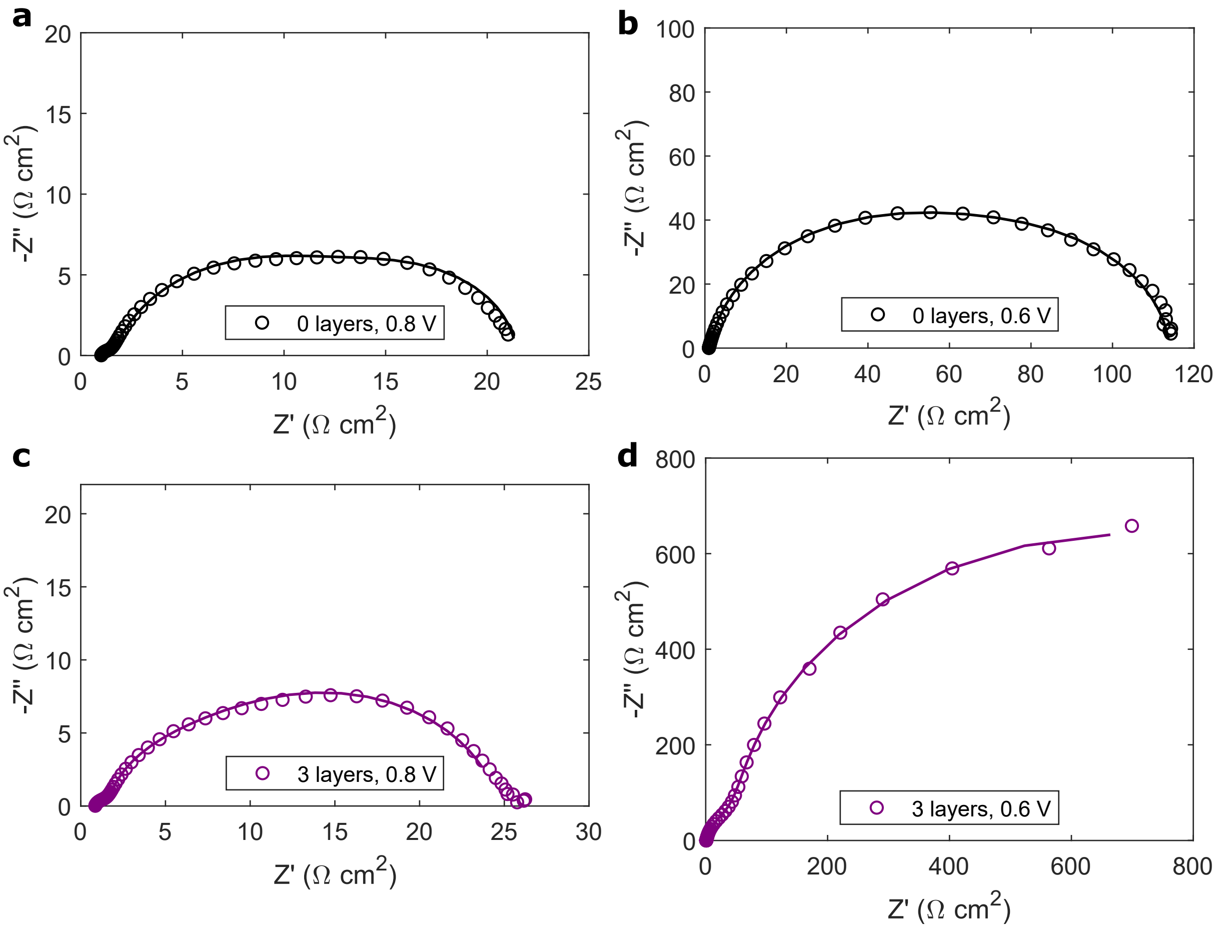

Figure S8: Electrochemical impedance spectra of complete DSC devices under illumination; **a** and **b**, for devices lacking any blocking layer ('0 cycles'), and **c** and **d**, devices with the (here) optimal blocking layer thickness of three spray repetitions, at different potentials.

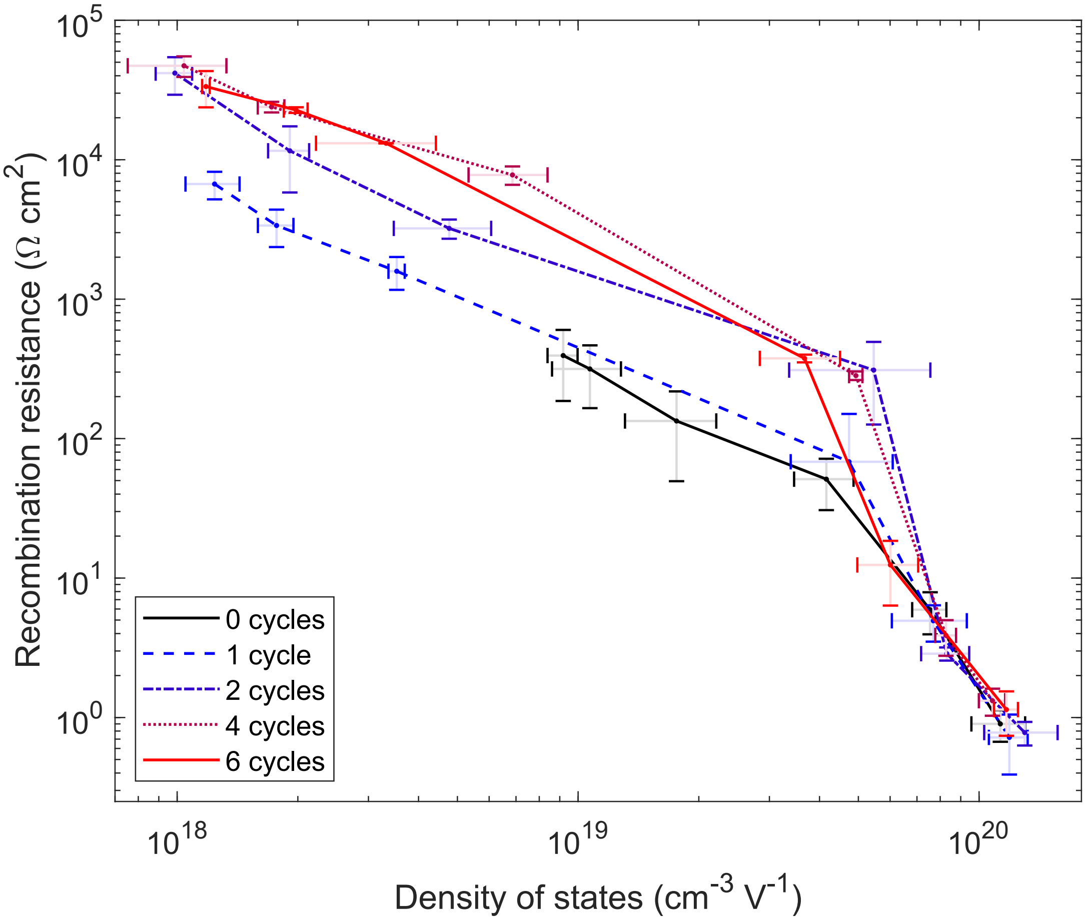

Figure S9: Electronic recombination resistance versus density of electronic states by blocking layer thickness from electrochemical impedance analysis of full DSC devices.

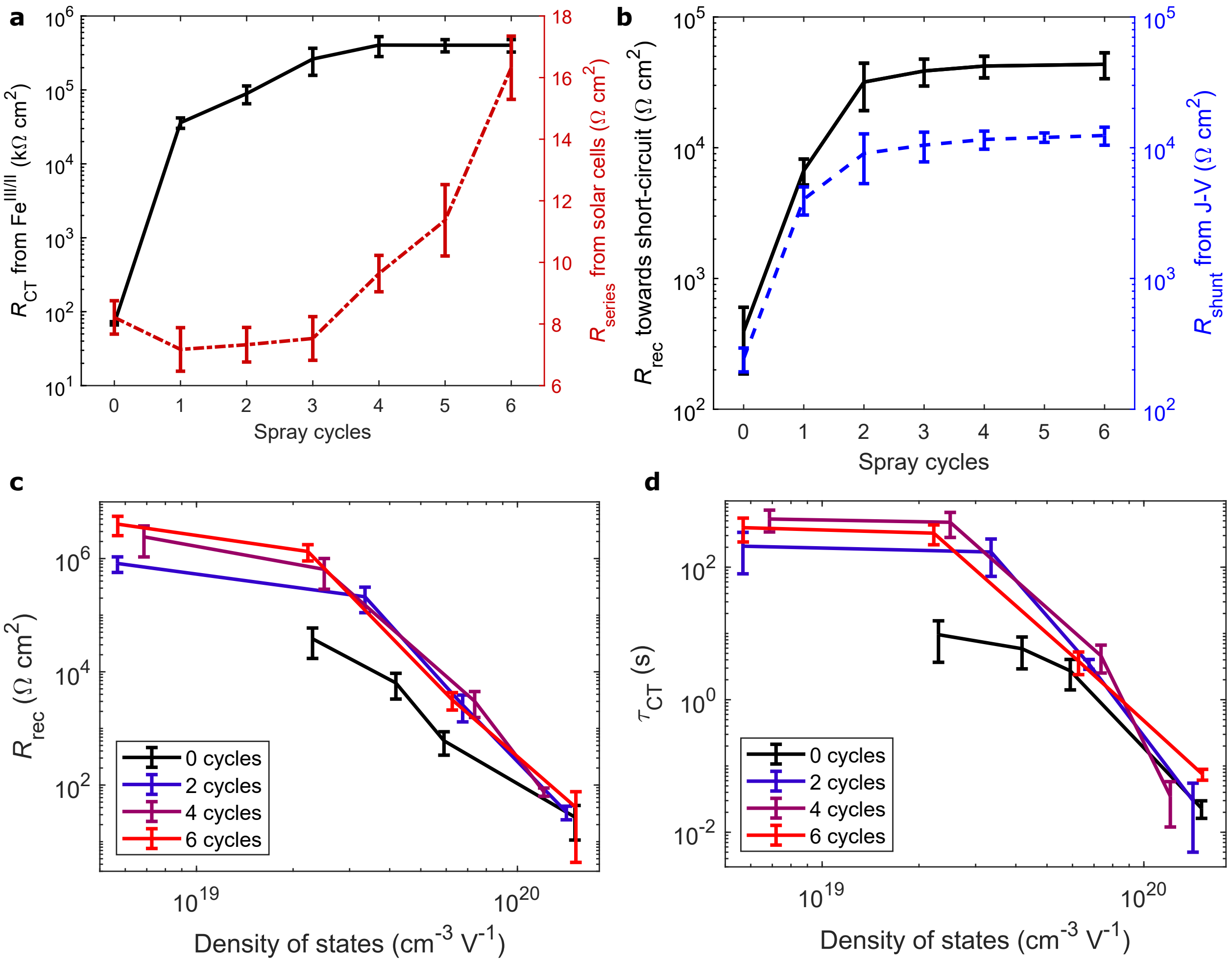

Figure S10: **a**, Charge transfer resistance and series resistance versus blocking layer thickness. **b**, Recombination resistance from electrochemical impedance spectroscopy and shunt resistance versus blocking layer thickness. **c**, 'Recombination' resistance across the m-TiO_2|dye|electrolyte interface in dark versus the density of states. **d**, Time constant $\tau = R_{rec}C_{rec} = (R_{rec}Q_{rec})^{\beta}$ in dark versus the density of states.
